# Investigating the impact of temperature on growth rate of the root rot fungus, Gymnopus fusipes

**DOI:** 10.1099/acmi.0.000738.v3

**Published:** 2024-06-25

**Authors:** Bethany J. Pettifor, Anparasy Kajamuhan, Sandra Denman, James E. McDonald

**Affiliations:** 1 School of Natural Sciences, Bangor University, Deiniol Road, Bangor, Gwynedd, LL57 2UW, UK; 2 Forest Research, Centre for Forestry and Climate Change, Alice Holt Lodge, Farnham, Surrey GU10 4LH, UK

**Keywords:** *Collybia*, growth rate, *Gymnopus fusipes*, oak, root rot, temperature

## Abstract

*Gymnopus fusipes* is an understudied root rot pathogen associated with multiple tree species and is linked to episodes of oak decline across the United Kingdom and Europe. Although the reported distribution of *G. fusipes* is broad, many observations rely solely on visual identification of fruiting bodies, which can be unreliable, and lack confirmation by molecular and/or isolation data to verify this broad ecological range. Given the paucity of information regarding the true ecological distribution of * G. fusipes*, it is difficult to predict and model the potential distribution of the species under both current and future climate scenarios. In this study, to determine the growth capabilities of *G. fusipes* across a range of ecologically relevant temperatures*,* five geographically diverse isolates of *G. fusipes* were grown at five different temperatures ranging from 4–37°C, to determine the optimal temperature for *G. fusipes* growth, and to establish whether geographically diverse isolates exhibit local adaptation to temperature tolerance. Incubation temperature had a significant effect on *G. fusipes* growth rate, with 25°C representing the optimum (*P*<0.001). Isolates had differing growth rates at each of the temperatures, with an isolate from the UK having the highest overall growth rate across all five temperatures tested (*P*<0.001), and at the optimum, increased by a mean value of over 4915 mm^2^. Local adaptation to temperature tolerance was not found in the isolates tested. These data demonstrate the optimal incubation temperature for future laboratory studies on *G. fusipes* and provide the first data on the growth rate of this pathogen across ecologically relevant climate ranges that may inform land managers, modellers, and policy makers in predicting the current and potentially future geographical limits of this widespread root rot pathogen.

## Data Summary

Data underpinning this research can be found in the Supplementary File (File S1: Growth Rate Results) with the online version of this article.

## Introduction

*Gymnopus fusipes* (Bull.:Fr.) Grey (syn. *Collybia fusipes*) is an understudied primary fungal pathogen, responsible for causing root rot on a number of economically important tree hosts, mainly oak, across the UK and Europe [1,7]. *G. fusipes* is an agaricomycete fungus situated in the family *Omphalotaceae* [8], with the genus *Gymnopus* consisting of around 300 plant-associated species distributed almost globally [9]. Since its identification as a primary pathogen in the mid-1980s, *Gymnopus fusipes* has been linked with numerous episodes of oak decline in the UK and Europe [10,11]. Though confirmed distribution, based on molecular analysis or fungal isolation is limited to the UK and Europe, less conclusive methods suggest a wider range, potentially encompassing Europe, America, Asia and northern Africa [12,16]. *G. fusipes* is reported to be the cause of root rot in a number of oak species (*Quercus* L.), as well as being present on beech (*Fagus* L.), hornbeam (*Carpinus* L.), chestnut (*Castanea* L.) [5,17] and silver fir (*Abies alba* Mill.) [18].

*Gymnopus fusipes* root infection is evident at below ground-level as orange lesions on the main roots of the host tree, with white mycelia scattered throughout and black cord-like structures close to the bark surface [19]. Trees infected with *G. fusipes* often go undiagnosed due a frequent lack of visible symptoms, such as fruiting bodies and a deteriorating crown condition, even with a severe infection [17,19]. In order to confirm infection in the absence of fruiting bodies, buttress roots must be exposed, and destructive sampling carried out. Thus the lack of disease detection, coupled with the destruction of the large anchoring roots, results in both young and mature trees being at a higher risk of being wind thrown [20]. While infection with *G. fusipes* is a slow process, taking up to 30 years from infection to mortality of the host [21], it can be devastating, destroying large central roots, and often whole root systems, leading to an increased risk to habitats, people and property due to the increased potential for falling trees [19].

Climatic factors, such as temperature, rainfall, and extreme weather considerably influence various ecological processes, ecosystem services and biodiversity [22,23]. A changing climate, and specifically a change in temperature, can have both direct and indirect effects on the distribution and activity of forest pathogens, due to the multitude of complex temperature-sensitive biological processes involved in infection success and host/pathogen survival [24]. Features of pathogen biology such as growth, reproduction and dispersal can be directly affected by changes in temperature. Indirect effects of temperature, such as changes in host distribution, or sub-optimal host conditions, can cause stress and increase susceptibility to infection [25]. An example of this can be seen in *Dothistroma pini*, a causal agent of *Dothistroma* needle blight (DNB) in France, where a gradual increase in mean temperature was identified as the key factor explaining the recent increased prevalence of the pathogen. *Dothistroma pini*, which although present in the country for a number of decades, had previously been unable to exist further north due to its need for a warmer climate [26,27].

Much uncertainty surrounds how pathogens and tree hosts will respond to a changing climate, partly due to a lack of empirical data on their tolerances and limits to different climatic elements [25]. This is made more difficult due to the fact that although fungal species can survive at a wide range of temperatures, their optimal growth rate and metabolic processing can require a much narrower margin, even when other factors, such as nutrient availability, remain constant [25,28]. Local adaptation to specific climates can cause geographic variation in virulence, evident in forest pathogens where isolates from different locations react differently to temperature. For example, in the case of *Phytophthora infestans*, isolates from warmer climates were found to be less virulent at colder temperatures, and similarly, isolates from colder climates were less aggressive under warmer temperatures, with temperature differences for peak aggressiveness between isolates being up to 4°C [29]. This can be explained by genetic differentiation, developing from local adaptation to environmental variables, which leads to a trade-off between enzyme stability and function, with those optimized for high temperatures being less effective at low temperatures and vice versa [30]. *Puccinia striiformis* f. sp. *tritici,* the causal agent of stripe rust (yellow rust) on wheat, previously known to prefer cooler climates, has in recent years become more prevalent in warmer areas including the eastern USA and Australia [31]. These isolates appear to not only have evolved to survive at these higher temperatures, thus increasing the pathogen’s distribution, but have also been found to be much more aggressive at the higher temperature across numerous variables when compared to the isolates from cooler climates, including growth rate (88% increase), lesion size (50% wider) and spore production (370% more spores) [32].

The effects of temperature on growth rate has not been researched in *G. fusipes*, other than one study that utilized predictive species distribution modelling, herbarium data, and 75 environmental predictor variables, which determined temperature to be a limiting factor for the spread of *G. fusipes* in Norway, where *G. fusipes* did not reach the northern limits of the *Quercus* host range [33]. However, the effect of temperature on the growth rate of *G. fusipes* is unknown, and would be beneficial to determine if temperature has any distribution limiting effects or effects on virulence in this species. Waterlogging has previously been identified (and well-studied) as a limiting factor of *G. fusipes*, and is known to affect the survival of *G. fusipes* inoculum and infection success, due to the intolerance of the species to hypoxia [5,17, 21, 34, 35].

The absence of empirical temperature-related data on *G. fusipes* along with sometimes spurious distribution data in the existing literature makes it difficult to determine the distribution of this species. By gaining an understanding of how *G. fusipes* growth responds to different temperatures, an estimation of the current distribution may be obtained. This data (along with other environmental factors such as response to waterlogging) are crucial in predicting the current and potential future distribution of *G. fusipes*. Being able to use this data for targeted diagnostics and monitoring of the species would be beneficial in order to identify potentially infected host trees before mortality, where there is a high risk of falling trees, which could cause damage to habitats, people, property, and could also affect revenue in plantations.

Therefore, the aim of this study was to firstly identify the optimal growth temperature of *G. fusipes* across a range of ecologically relevant temperatures, using a culture-based approach, and secondly to determine whether isolates from different geographical origins were locally adapted to temperature tolerance, using culture-based data and statistical modelling. This study aims to address the following hypotheses: (i) temperature will have a significant effect on the growth rate of *G. fusipes* isolates, and (ii) there will be no interaction between isolate and temperature that could suggest geographically diverse isolates have localized temperature adaptations.

Although this body of work focuses on *G. fusipes* growth in a laboratory setting on artificial media, it is known that hyphal growth rates underpin all fungal activity, including germination and fruiting body development [36]. Empirical data on hyphal growth rates can therefore inform models that explore distribution changes and virulence behaviours in a changing climate.

## Methods

### Obtaining isolates of *G. fusipes* and culture maintenance

At the time of the study, *G. fusipes* was not present in global culture collections, and direct isolation of this species in previous studies had been limited. Therefore, the only strains available for this experiment were those isolated during this study in the UK (AH1 and GMW83) and those previously isolated and provided by a research group in France (C41, C49, C52).

**Fig. 1.**
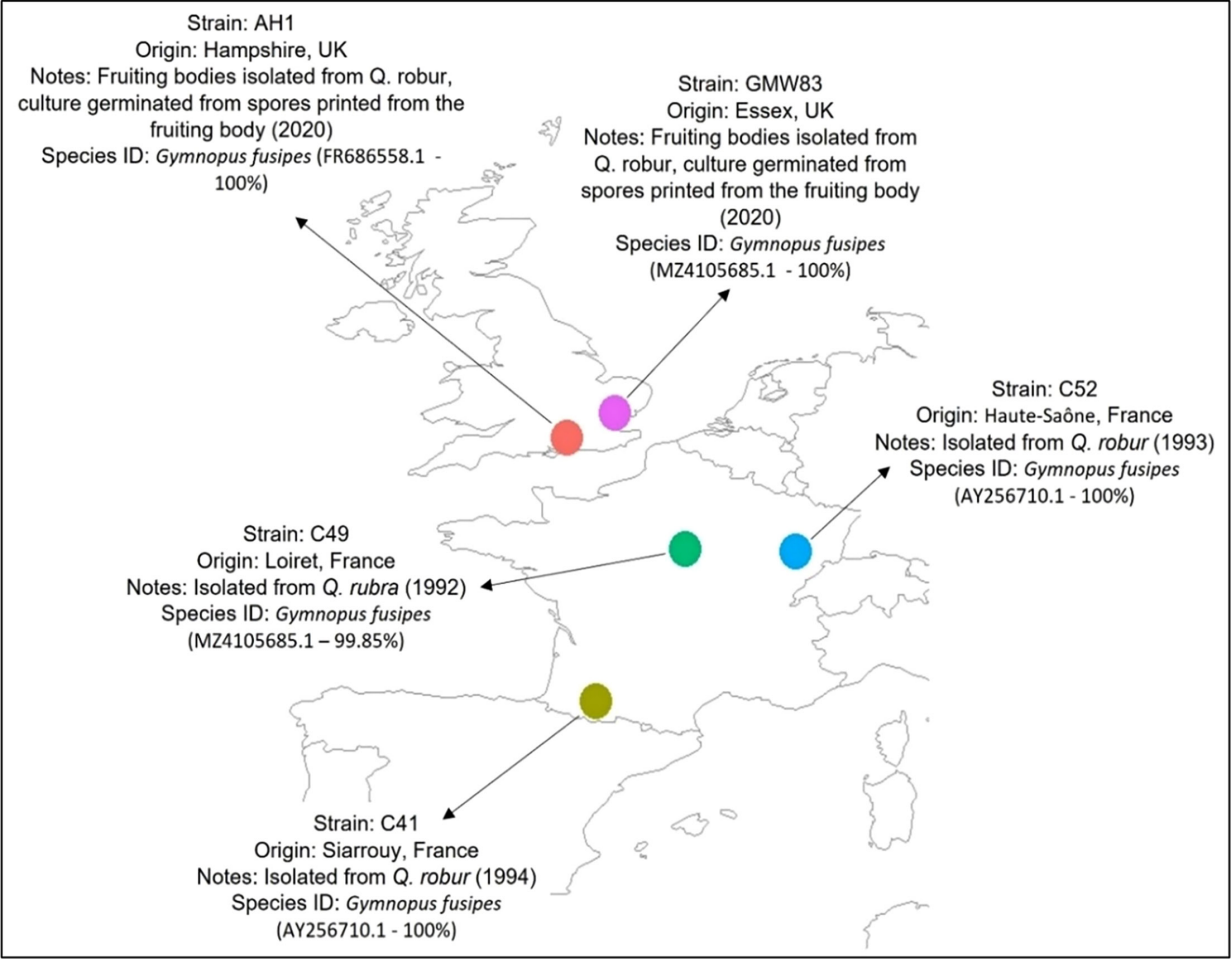
Location and information on the five isolates of *G. fusipes* that were used in this study. Strain name, place of origin and notes on isolation are detailed (along with blast search result identity accession number and similarity percentage for species identification).

Strains of *G. fusipes* were obtained from two areas of the UK and three regions of France (Fig. 1). Throughout the study, isolates were maintained on half strength malt extract agar [½ MEA: 25 g l^–1^ malt extract agar (Merck), 25 g l^–1^ Technical Agar (Oxoid), pH 5±2] at ambient room temperature (20–23°C).

### DNA extraction from *G. fusipes* isolates

DNA was extracted from *G. fusipes* strains using the MP Biomedicals FastDNA Spin Kit with a modified protocol. Briefly, 200–300 mg of *G. fusipes* mycelial tissue (double the quantity of tissue suggested in the manufacturer’s protocol), was added to a lyzing matrix A tube (containing a single 5 mm ceramic bead and garnet shards, MP Biomedicals), before adding 1 ml of CLS-Y and being homogenized a total of three times at 3 700 m s^–1^ for 40 s in a PowerLyzer24 instrument (Qiagen), the tubes were then centrifuged at 14 000 ***g*** for 10 min. From this point onwards all centrifugation steps were conducted at 14 000 ***g*** for 2 min. The supernatant (around 750 µl) was removed and transferred to a new 1.5 ml Eppendorf tube, and 750 µl of binding matrix was added. After inverting the tubes 10 times, tubes were secured to an orbital shaking platform and gently agitated (RPM 150) at room temperature for 5 min. After this, approximately 750 µl of the mixture was transferred to a SPIN Filter and centrifuged, and the flow through was discarded. The remaining 750 µl of the solution was transferred to the same SPIN Filter and again, centrifuged, the flow through discarded. Five hundred microlitres of prepared SEWS-M was added to the top of the SPIN Filter and the pellet above the filter was resuspended by pipetting up and down 10 to 20 times. The SPIN Filter tubes were centrifuged, the flow through discarded. The tubes were centrifuged again, before replacing the catch tube with a clean 1.5 ml Eppendorf tube. The pellet above the filter was resuspended in 100 µl of DES and tubes were incubated at 55°C for 5 min. After incubation, tubes were centrifuged to elute the DNA. Extracted DNA was purified using the Zymo Clean and concentrate kit (Zymo Research) following the manufacturer’s standard protocol ‘genomic DNA’.

### Confirmation of *G. fusipes* identity through PCR and sequencing of housekeeping genes

Using the purified DNA extracts, three housekeeping genes, the fungal internal transcribed spacer locus (*ITS*), the small ribosomal subunit (*18S rRNA*) and the translation elongation factor EF-1 alpha (*TEF1*), were amplified via PCR and sequenced, using Sanger sequencing, to confirm identity. Briefly, after vortex mixing, 1 µl of extracted and cleaned DNA was added to GoTaq Green Master Mix (Promega), in a PCR reaction as follows. Each reaction was 50 µl in volume, and along with the 1 µl of DNA template, contained 25 µl of 2 x GoTaq Green Master Mix, 22 µl of PCR-grade water, 1 µl each of 10 pmol of forward and reverse primers. Primer details and PCR cycling conditions are stated in Table 1.

**Table 1.** Primer details and cycling conditions for PCR amplification of the ITS, 18SrRNA and TEF1 gene regions

Gene region	Forward primer(5’ – 3’)	Reverse primer(5’ – 3’)	Cycling conditions	Reference
***ITS***	**ITS1:** TCCGTAGGTGAACCTGCGG	**ITS4:** TCCTCCGCTTATTGATATGC	Initial Denaturation: 95°C - 2 mins35 cycles of;Denaturation: 95°C - 30 s, Annealing: 55°C - 30 s,Extension: 72°C - 10 s,Final extension: 72°C - 5 min	[49]
***18S rRNA***	**NS1:** GTAGTCATATGCTTGTCTC	**NS8:** TCCGCAGGTTCACCTACGGA	Initial Denaturation: 95°C - 2 mins35 cycles of;Denaturation: 95°C - 30 s, Annealing: 55°C - 30 s,Extension: 72°C – 1 min,Final extension: 72°C - min	[49]
***TEF1***	**EF1-983F *(tef1f):***TACAARTGYGGTGGTATYGACA	**EF1-1567R *(tef1R)*:**ACNGACTTGACYTCAGTRGT	Initial Denaturation: 95°C - 3 mins35 cycles of;Denaturation: 94°C - 40 s, Annealing: 54°C - 45 s,Extension: 72°C – 1 min,Final extension: 72°C - 10 min	[50]

Ten microlitres of the PCR product was then visualized using a 1% agarose gel electrophoresis at 100V for 45 mins. One microlitre of PCR product was used for quantification using the Qubit dsDNA HS Assay Kit. The unpurified PCR product was sent to GENEWIZ (GENEWIZ, Takeley, UK) for Sanger sequencing. The resulting sequences were analysed in Geneious Prime (version 2023.0.4), where sequences were aligned, manually quality clipped, and realigned. The quality-controlled sequences were fed into the ‘Nucleotide blast’ programme on the NCBI blast database [National Center for Biotechnology Information (NCBI)]. *G. fusipes* isolates were confirmed by analysing the resulting *ITS*, *18S rRNA*, and *TEF1* gene sequences, whereby a sequence similarity of 97 % was used as a cut off for species delineation [37]. Each of the five isolates returned a similarity to *G. fusipes* of 99% or above, therefore were considered with confidence to be *G. fusipes*.

### *G. fusipes* inoculum production and experimental media preparation

The same growth temperature experiment, was conducted between 03.06.21 and 01.07.21, simultaneously at two different sites: Bangor University, UK, and Forest Research, Alice Hold Lodge, UK.

Prior to the start of the experiment, starter cultures of *G. fusipes* were prepared by filling sterile 90 mm Petri dishes with 40 ml of half-strength MEA (prepared as above). Agar plugs (5 mm in diameter) of each isolate (C41, C49, C52, AH1 and GMW83), were inoculated three per plate onto 10 agar plates, resulting in 30 plugs per isolate. These starter cultures were incubated at 25°C for 14 days.

After the 14 day incubation period, plates for the experiment were prepared by producing 150 sterile 90 mm petri dishes, each filled with 40 ml of ½ strength MEA, using a Fisherbrand bottle top dispenser. Perpendicular axes were drawn onto the bottom of the plates using a permanent marker.

### Identifying growth temperature optimum for *G. fusipes*

In order to support statistical robustness in the experiment, five biological replicates for each isolate under each temperature condition were used, meaning that a total of 25 plates per isolate were produced, by placing a mycelial disc in the centre of the perpendicular axis. Five biological replicates of the non-inoculated plates were also used at each temperature, and were kept for the duration of the experiment as non-inoculated negative controls to confirm the absence of contamination in the experimental process. After drawing around the circumference of the inoculum plug (5 mm in the Bangor University experiment, and 10 mm in the Forest Research experiment) with a thin black permanent marker (day 0 measurement), all plates were incubated at 25°C for 7 days, as an acclimatisation period.

After the 7 day acclimatization period, the plates were removed from the incubator, and the circumference of the growth was outlined (day 7 measurement). At this point, five replicates of each strain were placed in different incubators maintained at five different temperatures, 4°C, 10°C, 20°C, 25 and 37°C. The layout of the replicates in each of the incubators was completely randomized, using a random number generator, in order to avoid any effects of incubator positioning. The 30 plates (five replicates of each of the five strains, and five non-inoculated controls) were arranged in two layers of 15, and this layout was consistent across the five different incubation temperatures, and at both sites.

Isolates were incubated at these temperatures for a further 21 days (total 28 days), with the colony circumference being outlined on days 14, 21 and 28. After the experiment, measurements were taken along each of the axis on the base of the plate, giving a total of four radial measurements per plate. Data was compiled into an Excel spreadsheet for further analysis (File S1, available in the online version of this article).

### Data analysis

Firstly, the difference in radius between day 7 and day 28 of the experiment was calculated, to give the overall growth of each replicate in the experimental period, excluding the initial 7 day acclimatization period at 25°C. The four radial measurements for each experiment isolate replicate, at each time point, were converted into an estimate of colony area (using πr^2^), and the mean colony area was fitted to a linear model in order to visualize trends more easily. Separate plots were made for the two parallel independent experiments at Bangor University and Forest Research.

As the trends in the growth data were very similar across the two sites, the two datasets of colony area growth over the experimental period (day 7 to day 28) were then combined. After fitting the data to generalized linear mixed-effects models, initially considering temperature as a fixed effect, with isolate and the site where the experiment was conducted (Bangor University or Forest Research) as random effects, in order to determine the effect of temperature on growth of *G. fusipes*. Subsequently, the parameters were altered to consider the *G. fusipes* isolate as a fixed effect, to determine whether the isolate had an effect on growth rate, regardless of the temperature considered. Finally, the model was fitted to include the interaction between isolate and temperature. As the data presented a Gamma distribution, and was a measurement of area, this was addressed in the family link section of the model parameters, whereby Gamma family with a ‘sqrt’ link was determined for each version of the model. Statistical significance for each of the models were calculated using Chi squared tests. All statistical analyses were performed in R Studio (version 2022.02.3), with a confidence level of 95% required for statistical significance.

## Results

### Measuring the impact of temperature on the growth rate of *Gymnopus fusipes*

**Fig. 2.**
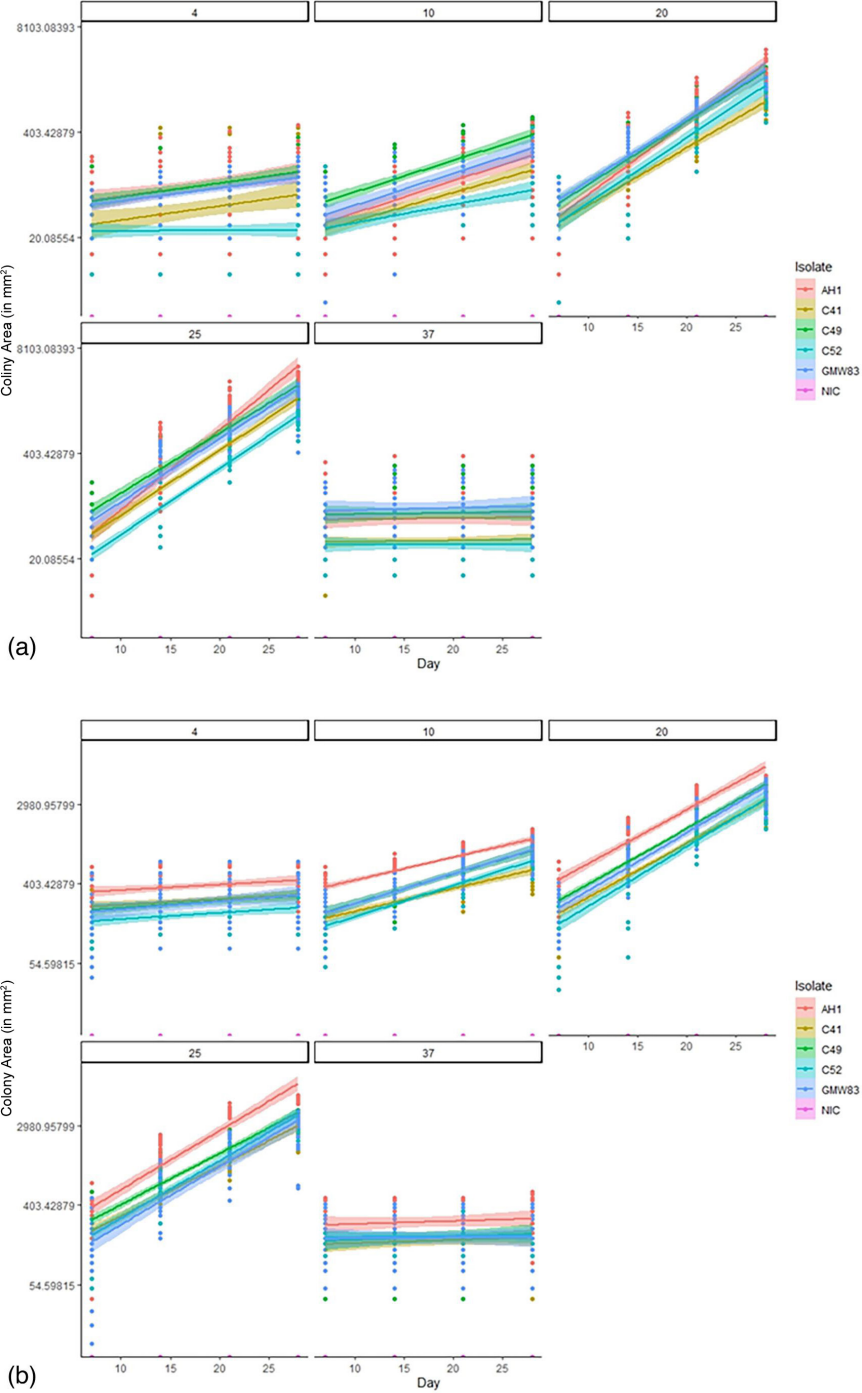
Colony area (mm^2^) of the *G. fusipes* isolates recorded between day 7 and day 28 of the growth rate experiment, 95% confidence intervals are indicated by the shaded area behind the line. (**a**) Measurements taken at Bangor University. (**b**) Measurements taken in the parallel experiment at Forest Research.

Fig. 2 shows the colony area (mm^2^) for *G. fusipes* isolates at each of the five tested temperatures.

The trends in both data sets were very similar, with all isolates undergoing a clear increase in colony area at 10, 20 and 25°C with the largest increase being at 20 and 25°C. Colony area increase at 4 and 37°C was much less clear. The mean difference in colony area between day 7 and day 28 for each of the isolates was calculated (including data from both sites) to provide a mean area increase for each of the experiment replicates over the experimental period. Over the 28 day experimental period, the amount of growth of *G. fusipes* isolates varied greatly between the different incubation temperatures, with area ranges of 0–402.6 mm^2^ at 4°C, 8.0–1 003.2 mm^2^ at 10°C, 703.3–5 400.2 mm^2^ at 20°C, 671.9–5 568.5 mm^2^ at 25°C and 0–86.2 mm^2^ at 37°C.

Across the five temperatures, *G. fusipes* isolate AH1 had the largest growth over the 28 day experiment, growing between 21.2 and 185.3 mm^2^ at 4°C, between 106.6 and 1003.2 mm^2^ at 10°C. At the higher temperatures of 20 and 25°C, isolate AH1 had a growth of 1 728.3–5 400.2 mm^2^ and 2 430.0–5 568.5 mm^2^, respectively. At 37°C, isolate AH1 still maintained the largest growth rate of the five isolates, growing between 0 and 86.2 mm^2^ over the course of the experiment.

### Determining statistical significance of temperature on *G. fusipes* growth rate

The results of the Chi squared test determining whether incubation temperature had a considerable effect on growth rate were significant (*P*<0.001), indicating that incubation temperature does have an effect on the growth rate of *G. fusipes*, with 25°C having the largest effect (*P*<0.001), and therefore being considered the optimal temperature for *G. fusipes* growth of those tested in this experiment.

### Determining statistical significance of isolate on *G. fusipes* growth rate

**Fig. 3.**
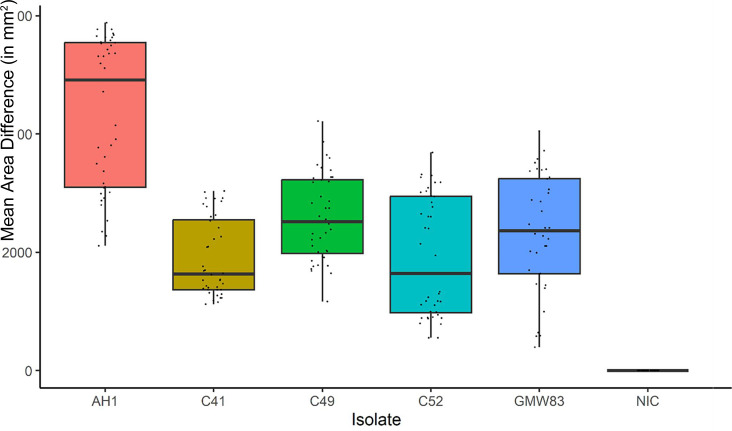
Mean area increase of the five *G. fusipes* isolates at 25°C. Isolate AH1 had a significantly larger increase in mean area size than the other isolates tested in the experiment at this optimal temperature (*P*<0.001).

The results of the Chi squared test determining whether the isolate had an effect on the growth rate was also significant (*P*<0.001), with isolate AH1 having the fastest growth rate out of the five isolates tested, regardless of the temperature being observed. The data highlighted that at 25°C, the optimal temperature for growth, isolate AH1 had the highest increase in mean area size over the experiment, with an overall mean increase (across the total ten replicates, five from BU and five from FR) of approximately 4915 mm^2^ (Fig. 3).

### Determining statistical significance of the relationship between temperature and isolate on *G. fusipes* growth rate indicative of localized adaptation to temperature tolerance

**Fig. 4.**
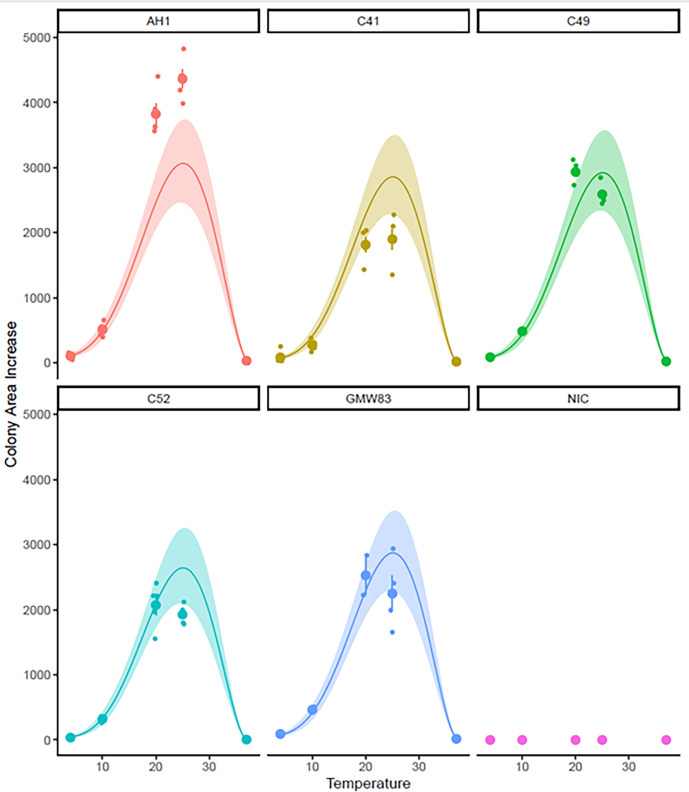
Effect of the *G. fusipes* isolate, and temperature on the colony area increase of *G. fusipes* in the experimental period (with mean and standard error indicated), a generalized linear mixed-effects model (with temperature as a polynomial number) has been fitted to the data and is indicated by the continuous line along with the 95% confidence intervals for the model.

The five isolates experienced different growth rates across the incubation temperatures (Fig. 4), with AH1 having the largest growth rate overall, and isolates C49, C52 and GMW83 maintaining an intermediate level of growth, and isolate C52, which had overall the smallest change in area size over the experimental period. Chi squared tests to determine the significance of the relationship between isolate and incubation temperature on the growth rate of *G. fusipes* was insignificant.

## Discussion

There is a severe lack of empirical data on the ecology and growth rate of *G. fusipes*, with limited culture-based analysis having been completed. The study represents the first instance of growth rate being investigated in *G. fusipes* at a range of temperatures. The main aim of this study, therefore, was to initially determine the optimal growth temperature for *G. fusipes in vitro*, using culture-based methods, and secondly to determine whether geographically diverse *G. fusipes* isolates showed signs of local adaptation to temperature tolerance, using culture-based data and statistical modelling. The data obtained from this experiment have addressed the following two hypotheses: (i) incubation temperature will have a significant effect on the growth rate of *G. fusipes* isolates, and (ii) geographically diverse isolates will not display signs of any local adaptation to varying temperatures.

Using generalized linear mixed-effects models and Chi squared tests, a significant effect (*P*<0.001) on fungal growth rate was found when incubating the *G. fusipes* isolates at different temperatures. This indicates that incubation temperature alone is statistically significant in its effects on the growth of *G. fusipes*, regardless of the isolate in question. The optimal incubation temperature for *G. fusipes* growth, of the five temperatures tested, was found to be 25°C, having the largest influence on the growth rate across all five of the isolates. Isolate AH1 was found to have the fastest growth rate of all five *G. fusipes* isolates tested, regardless of the incubation temperature (*P*<0.001), with isolates C49, GMW83 and C41 having a similar, intermediate rate of growth, and isolate C52 having the slowest rate of growth overall. The analysis to determine whether geographically diverse isolates would display signs of any local adaptation in temperature tolerance was statistically insignificant, meaning that in this case there was no relationship between the isolate origin and the optimum growth temperature.

The data obtained in this study determined the optimal growth temperature for *G. fusipes* is 25°C, which does not necessarily correlate with the climate data for the UK and France. In the south of the UK (the origin of isolates AH1 and GMW83), the temperature reaches around 22°C in the peak of summer, 3°C lower than the 25°C optimum. In France (the origin of isolates C41, C49 and C52), the temperature in the summer reaches around 24°C, which is closer to the 25°C optimum [38]. This indicates that *G. fusipes* isolates, particularly in the UK, may be growing slower than isolates suggested to occur in warmer climates, such as Italy and Greece [16,18, 39].

This experiment has provided empirical data that could potentially be used in processes such as predictive modelling, which could help to determine the current and potential future distribution of *G. fusipes*. This has been completed for other root rot pathogens, including *Phytophthora cinnamoni* Rands., the cause of ink disease on European chestnut, as well as numerous others, where temperature data was combined with other environmental response data to predict potential future distributions [40]. Environmental niche modelling, or species distribution modelling are key tools providing analysis of biotic and abiotic factors such as environmental conditions and host availability, to identify potential distribution of a species, which can be used to determine effects of a varying factors including climate change [41]. Although this can be considered a crucial task in the understanding of the spread of phytopathogens, the majority of environmental niche modelling has been done on plants, animals and insects [42]. It has been suggested that the low number of phytopathogen environmental niche models could be due to the difficult nature of phytopathogen study, including identification difficulty, lack of visible symptoms, and life-cycle effects, as well as the lack of available data on factors such as global distribution [43]. In order to utilize existing modelling software, such as CLIMEX, to determine the potential distribution of a phytopathogen such as *G. fusipes*, numerous environmental factors must be considered. These parameters include moisture, heat and cold stress, dry and wet stress, and cold–wet stress, as well as temperature [44].

Although the second hypothesis [(ii) geographically diverse isolates will not display signs of any local adaptation to varying temperatures] was not supported by this study, phenotypic variation in temperature tolerance and sensitivity is well documented in a number of other fungal plant pathogens, including *Puccinia striiformis*, the causal agent of leaf rust in wheat plants [45,46], *Phytophthora infestans,* the cause of potato late blight [45,47], and *Mycosphaerella graminicola*, the cause of leaf blotch of wheat [30]. In the case of *G. fusipes*, a number of factors could potentially explain the lack of findings. For example, there was an absence of isolates in global culture collections, meaning that the study was limited to isolates from the UK and France. The study would benefit from obtaining isolates from more varied climatic zones, more representative of the transcontinental distribution of *G. fusipes*.

Ideally, the growth rate of *G. fusipes* would have been measured in an infected root system by assessing lesion size, and not on agar plates, in order to provide a realistic expectation for growth rate, however this is not necessarily feasible for this pathogen. *G. fusipes* infection occurs below the ground level, meaning that it would be difficult to make continual measurements without disrupting the infection system. *G. fusipes* infection is difficult to identify [35], and more difficult to determine infection severity without unrooting the tree host, therefore it would be unwise to attempt to directly measure growth rate in naturally occurring infections, due to the inherent variability of the system. In artificial inoculation experiments, * G. fusipes* can take between 6 months and 2 years [20,21] to develop lesions that can be measured. Measuring growth *in Planta* has been conducted in some tree pathogens, including some species from the genus *Phytophthora*, that are known to infect species of *Rhododendron* [48], however these are fast growing leaf pathogens, and as such are more accessible for continued measurements of growth.

Future research on *G. fusipes* should aim to prioritize obtaining empirical data regarding the effect of various environmental factors on *G. fusipes*, in order to inform distribution modelling. This could include repeating this experiment with more temperature increments between 25 and 37°C, to determine the temperature limits. An effort should also be made to increase accurate identification of *G. fusipes* in the field, on a global scale, as this will provide evidence to support the somewhat questionable distribution claims made about this species. It is important that a deeper understanding of the effects of a changing climate on this species is acquired, as particularly an increase in temperature and a decrease in waterlogging occurrences may have a significant effect on the spread and also aggressiveness of this pathogen.

## supplementary material

10.1099/acmi.0.000738.v3 Uncited Supplementary Material 1.

## Abbreviations

DNB: dothistroma needle blight
ITS: internal transcribed spacer
MEA: malt extract agar

## References

[1] Aguayo J, Husson C, Chancerel E, Fabreguettes O, Chandelier A (2021). Combining permanent aerobiological networks and molecular analyses for large‐scale surveillance of forest fungal pathogens: A proof‐of‐concept. Plant Pathology.

[2] Boddy L, Thompson W (1983). Decomposition of suppressed oak trees in even‐Aged plantations: I. Stand characteristics and decay of aerial parts. New Phytol.

[3] Chandelier A, Hulin J, San Martin G, Debode F, Massart S (2021). Comparison of qPCR and metabarcoding methods as tools for the detection of airborne inoculum of forest fungal pathogens. Phytopathology.

[4] Marcais B, Martin F, Delatour C (1998). Structure of *Collybia fusipes* populations in two infected oak stands. Mycol Res.

[5] Piou D, Delatour C, Marçais B (2002). Hosts and distribution of *Collybia fusipes* in France and factors related to the disease’s severity. Forest Pathology.

[6] Przybyl K (1994). *Collybiafusipes* [Bull.ex FR.] Quelet and oak decline in Poland: Saprophytic and parasitic forms of the fungus. Arbor Kórnickie.

[7] Schmidt O, Gaiser O, Dujesiefken D (2012). Molecular identification of decay fungi in the wood of urban trees. Eur J Forest Res.

[8] Ványolós A, Dékány M, Kovács B, Krámos B, Bérdi P (2016). Gymnopeptides A and B, cyclic octadecapeptides from the mushroom *Gymnopus fusipes*. Org Lett.

[9] Jang S, Jang Y, Lim YW, Kim C, Ahn BJ (2016). Phylogenetic identification of Korean *Gymnopus* spp. and the first report of 3 species: *G. iocephalus*, *G. polygrammus*, and *G. subnudus*. Mycobiology.

[10] Delatour C, Guillaumin JJ (1984). Un pourridie´ me´connu: le *Collybia fusipes* (Bull. ex FR.) Quel. Comptes Rendus des Seances l’Academie d’Agriculture Fr.

[11] Guillaumin J-J, Bernard C, Delatour C, Belgrand M (1985). Contribution à l’étude du dépérissement du chêne : pathologie racinaire en forêt de Tronçais. Ann des Sci For.

[12] Ben M, Ali HB, Aschi-Smiti S (2013). Mycocoenologic study of the macrofungi on the forest of Jbel Elbir (Aın Draham, Jendouba, Tunisia). Afr J Ecol.

[13] Gabel A, Ebbert E, Lovett K (2004). Macrofungi collected from the Black Hills of South Dakota and Bear Lodge Mountains of Wyoming. Am Midl Nat.

[14] Reverchon F, María del Ortega-Larrocea P, Pérez-Moreno J (2010). Saprophytic fungal communities change in diversity and species composition across a volcanic soil chronosequence at Sierra del Chichinautzin, Mexico. Ann Microbiol.

[15] Semwal KC, Bhatt VK (2019). A report on diversity and distribution of macrofungi in the Garhwal Himalaya, Uttarakhand, India. Biodivers Res Conserv.

[16] Pettifor BJ, Denman S, Mcdonald JE (2022). Using a systematic approach to synthesize existing knowledge on *Gymnopus fusipes* (Syn *Collybia fusipes*), the cause of *Collybia* root rot. For Pathol.

[17] Marçais B, Caël O (2000). Comparison of the susceptibility of *Quercus petraea*, *Q. robur* and *Q. rubra* to *Collybia fusipes*. Eur J Plant Pathol.

[18] Ambrosio E, Brotzu R, Lancellotti E, Franceschini A, Zotti M (2015). Macrofungi in *Abies alba* Miller plantation in north-western Sardinia, Italy. Micol Ital.

[19] Marçais B, Caël O, Delatour C (1999). Measuring the impact of *Collybia fusipes* on the root system of oak trees. Ann For Sci.

[20] Marçais B, Delatour C (1996). Inoculation of Oak (*Quercus robur* and *Q. rubra*) with *Collybia fusipes*. Plant Dis.

[21] Camy C, Delatour C, Caël O, Marçais B (2003). Inoculation of mature pedunculate oaks (*Quercus robur*) with the root rot fungus *Collybia fusipes*: relationships with tree vigour and soil factors. Eur J Plant Pathol.

[22] Grimm NB, Staudinger MD, Staudt A, Carter SL, Chapin FS (2013). Climate‐change impacts on ecological systems: introduction to a US assessment. Frontiers Ecol Environ.

[23] Stenseth NC, Mysterud A, Ottersen G, Hurrell JW, Chan KS (2002). Ecological effects of climate fluctuations. Science.

[24] Voyles J, Johnson LR, Rohr J, Kelly R, Barron C (2017). Diversity in growth patterns among strains of the lethal fungal pathogen *Batrachochytrium dendrobatidis* across extended thermal optima. Oecologia.

[25] Dukes JS, Pontius J, Orwig D, Jeffrey RG, Vikki LR (2009). Responses of insect pests, pathogens, and invasive plant species to climate change in the forests of northeastern North America: what can we predict?. Can J For Res.

[26] Desprez-Loustau M-L, Aguayo J, Dutech C, Hayden KJ, Husson C (2016). An evolutionary ecology perspective to address forest pathology challenges of today and tomorrow. Ann For Sci.

[27] Fabre B, Ioos R, Piou D, Marçais B (2012). Is the emergence of *Dothistroma* needle blight of pine in France caused by the cryptic species *Dothistroma pini*?. Phytopathology.

[28] Li Y, Wadsö L, Larsson L (2009). Impact of temperature on growth and metabolic efficiency of *Penicillium roqueforti*--correlations between produced heat, ergosterol content and biomass. J Appl Microbiol.

[29] Wu EJ, Wang YP, Yang LN, Zhao MZ, Zhan J (2022). Elevating air temperature may enhance future epidemic risk of the plant pathogen *Phytophthora infestans*. J Fungi.

[30] Zhan J, McDonald BA (2011). Thermal adaptation in the fungal pathogen *Mycosphaerella graminicola*. Mol Ecol.

[31] Nnadi NE, Carter DA (2021). Climate change and the emergence of fungal pathogens. PLoS Pathog.

[32] Milus EA, Kristensen K, Hovmøller MS (2009). Evidence for increased aggressiveness in a recent widespread strain of *Puccinia striiformis* f. sp. *tritici* causing stripe rust of wheat. Phytopathology.

[33] Wollan AK, Bakkestuen V, Kauserud H, Gulden G, Halvorsen R (2008). Modelling and predicting fungal distribution patterns using herbarium data. J Biogeogr.

[34] Camy C, Delatour C, Marçais B (2003). Relationships between soil factors, *Quercus robur* health, *Collybia fusipes* root infection and *Phytophthora* presence. Ann For Sci.

[35] Marçais B, Caël O (2001). Relation between *Collybia fusipes* root rot and growth of pedunculate oak. Can J For Res.

[36] Brasier CM, Worral J (1999). Structure and Dynamics of Fungal Populations.

[37] Pettifor BJ, Doonan J, Denman S, McDonald JE (2020). Survival of *Brenneria goodwinii* and *Gibbsiella quercinecans*, associated with acute oak decline, in rainwater and forest soil. Syst Appl Microbiol.

[38] Weather Atlas (2023). The weather around the World – List of countries. https://www.weather-atlas.com/en/countries.

[39] Diamandis S, Perlerou C (2001). The mycoflora of the chestnut ecosystems in Greece. Forest Snow Land Res.

[40] Desprez-Loustau M-L, Robin C, Reynaud G, Déqué M, Badeau V (2007). Simulating the effects of a climate-change scenario on the geographical range and activity of forest-pathogenic fungi. Can J Plant Pathol.

[41] Wang R, Li Q, He S, Liu Y, Wang M (2018). Modeling and mapping the current and future distribution of *Pseudomonas syringae* pv. *actinidiae* under climate change in China. PLoS One.

[42] Chaloner TM, Gurr SJ, Bebber DP (2020). Geometry and evolution of the ecological niche in plant-associated microbes. Nat Commun.

[43] Ireland KB, Kriticos DJ (2019). Why are plant pathogens under-represented in eco-climatic niche modelling?. Int J Pest Manag.

[44] Yonow T, Hattingh V, de Villiers M (2013). CLIMEX modelling of the potential global distribution of the citrus black spot disease caused by *Guignardia citricarpa* and the risk posed to Europe. Crop Prot.

[45] Castroverde CDM, He SY, Lansing E, Lansing E, Lansing E (2015). Plant and pathogen warfare under changing climate conditions.

[46] Park RF (1990). The role of temperature and rainfall in the epidemiology of *Puccinia striiformis* f.sp. *tritici* in the summer rainfall area of eastern Australia. Plant Pathol.

[47] Shakya SK, Goss EM, Dufault NS, van Bruggen AHC (2015). Potential effects of diurnal temperature oscillations on potato late blight with special reference to climate change. Phytopathology.

[48] Taylor CR, Grünwald NJ (2021). Growth, infection and aggressiveness of *Phytophthora* pathogens on *Rhododendron* leaves. *CABI Agric Biosci*.

[49] White TJ, Bruns T, Lee S, Taylor J (1990). PCR Protocols: A Guide to Methods and Applications.

[50] Morehouse EA, James TY, Gangley ARD, Vilgalys R, Berger L (2003). Multilocus sequence typing suggests the chytrid pathogen. Mol Ecol.

